# Differences between Elite Male and Female Badminton Athletes Regarding Heart Rate Variability, Arterial Stiffness, and Aerobic Capacity

**DOI:** 10.3390/ijerph19063206

**Published:** 2022-03-09

**Authors:** Ching-Chieh Tai, Yi-Liang Chen, Ludek Kalfirt, Kunanya Masodsai, Chia-Ting Su, Ai-Lun Yang

**Affiliations:** 1 Graduate Institute of Sports Training, University of Taipei, Taipei 11153, Taiwan; sguy062020@hotmail.com.tw (C.-C.T.); yiliang@utaipei.edu.tw (Y.-L.C.); 2 Institute of Sports Sciences, University of Taipei, Taipei 11153, Taiwan; kalfirtl77@gmail.com; 3 Faculty of Sports Science, Chulalongkorn University, Bangkok 10330, Thailand; kunanya.m@chula.ac.th; 4 Department of Occupational Therapy, College of Medicine, Fu Jen Catholic University, New Taipei City 24205, Taiwan; chiatingsu@gmail.com

**Keywords:** heart rate, blood pressure, arterial stiffness index, maximal oxygen consumption, anaerobic threshold, treadmill test, badminton, elite players

## Abstract

Cardiovascular health and aerobic capacity play crucial roles in determining the performance of athletes in the highly competitive sport of badminton. Few studies have directly compared heart rate variability (HRV), arterial stiffness, and aerobic capacity between male and female athletes, especially among badminton athletes. This study investigated sex differences in HRV, arterial stiffness, and aerobic capacity in badminton athletes. Elite badminton athletes were recruited and divided into male (*n* = 20, 21.0 ± 1.8 years old) and female (*n* = 16, 21.2 ± 2.3 years old) groups. Both groups performed an incremental treadmill running test for the evaluation of maximal oxygen consumption ($\dot{V}$O_2_max), anaerobic threshold, and time to exhaustion. They started exercising at a treadmill speed of 2.7 km/h and an inclination of 10% gradient for 3 min, and the speed and inclination were gradually increased every 3 min until they were exhausted or fatigued volitionally. HRV was examined using the Polar heart rate monitor over a period of 5 min at rest in the supine position. Subsequently, the index of arterial stiffness was examined under the same condition. Our results revealed significant differences between the male and female athletes in $\dot{V}$O_2_max (men: 60.38 ± 8.98 mL/kg/min, women: 48.13 ± 7.72 mL/kg/min, *p* < 0.05), anaerobic threshold (men: 41.50 ± 7.26 mL/kg/min, women: 32.51 ± 6.19 mL/kg/min, *p* < 0.05), time to exhaustion (men: 902.15 ± 120.15 s, women: 780.56 ± 67.63 s, *p* < 0.05), systolic blood pressure (men: 125.27 ± 7.76 mmHg, women: 107.16 ± 11.09 mmHg, *p* < 0.05), and arterial stiffness index (men: 63.56 ± 12.55, women: 53.83 ± 8.03, *p* < 0.05). However, no significant differences in HRV measures were observed between the two groups. These findings suggested that the male badminton athletes demonstrated significantly higher aerobic capacity than did the female athletes, but there were no significant differences in HRV measures. The female athletes exhibited superior arterial function, compared with their male counterparts.

## 1. Introduction

Badminton, a highly competitive sport, demands high aerobic and anaerobic capacity [1]. A badminton match is characterized by bouts of high-intensity intermittent exercise separated by brief periods of rest [2]. Hence, aerobic capacity is key to competitive performance. For elite male and female badminton athletes, their maximal oxygen consumption ($\dot{V}$O_2_max) should be >60 and >52 mL/kg/min, respectively [3]. In addition, male and female athletes with similar levels of competitive performance may present differences in $\dot{V}$O_2_max and body composition (e.g., body fat) [4]. However, few studies have examined differences in aerobic capacity between elite male and female badminton athletes. Understanding these sex differences might be beneficial to design effective training programs for improving sports performance in elite players.

Heart rate variability (HRV) is commonly used in clinical practice to estimate the physiologic status of cardiac autonomic activity. HRV is a widely used marker that reflects the interplay between the sympathetic and parasympathetic branches of the autonomic nervous system (ANS) and the modulation of normal cardiac rhythm [5,6]. Numerous studies supported the use of HRV for monitoring the adaptation and maladaptation during and after training to determine optimal training loads leading to improvements in sports performance [5,6,7,8]. Better HRV profiles have been associated with greater improvements in aerobic capacity and endurance performance. These profiles can be indicated by the higher values of the normal-to-normal (NN) intervals, standard deviation of the NN intervals (SDNN), and high-frequency (HF) power [9,10,11]. Moreover, several studies focusing on sex differences in HRV have reported that, compared with men, women exhibited increased parasympathetic and decreased sympathetic control of the heart rate [12,13]. Although men and women differ in some respects, long-term endurance training improved some HRV parameters in both genders [14,15]. However, few studies have focused on how male and female badminton athletes differ in HRV. A recent study indicated that the HRV-guided training was superior for enhancing vagal-related HRV indices, which could be related to greater endurance performance. When resting HRV was within or above baseline ranges, high-intensity exercise training was prescribed; otherwise, low-intensity exercise training was prescribed when HRV values were suppressed [16]. Gaining more understanding of HRV profiles in male and female athletes could provide appropriate training prescription and effective assessment of cardiovascular adaptation.

Arterial stiffness, which indicates early vascular changes, is a crucial predictor of cardiovascular risk and mortality [17]. Moreover, arterial stiffness is associated with several aspects of exercise performance, such as aerobic capacity. It may negatively affect aerobic capacity, partly by increasing pulse pressure, which alters myocardial work capacity and coronary perfusion [18]. In previous studies, healthy young men who underwent short-term endurance training (from 6 days to 8 weeks) exhibited lowered (i.e., better) arterial stiffness [19,20]. Studies, despite being few in number, have indicated the long-term benefits of endurance training (>6 months) for arterial stiffness in young individuals [21]. For athletes, overload training would result in increased resting arterial stiffness and reduced stroke volume during exercise, which negatively affects exercise performance [22]. To date, few studies have evaluated sex differences in arterial stiffness among athletes. Learning more about sex differences in arterial stiffness for athletes would be beneficial to avoid risk factors caused by overload training programs.

Exploring sex differences in cardiovascular health and aerobic capacity should be of concern for the design of effective training programs for men and women in either health promotion or sports performance. Currently, few studies have focused on direct comparisons of HRV, arterial stiffness, and aerobic capacity between male and female athletes, especially among badminton athletes. Thus, this study investigated gender differences in HRV, arterial stiffness, and aerobic capacity in elite badminton athletes. It was hypothesized that these parameters would differ between elite male and female badminton athletes.

## 2. Materials and Methods

### 2.1. Participants

**Figure 1:**
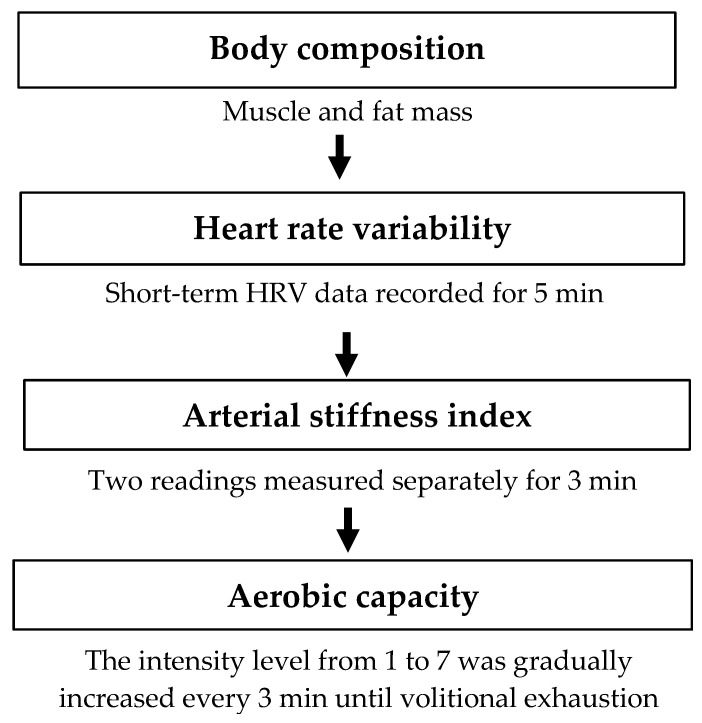
The order of the measurements in the present study.

The observational case–control design was used in this study. Elite badminton athletes in Taiwan aged between 20 and 28 years were recruited to this study (20 men and 16 women). The advertisements of this study were posted on the universities where training programs were provided for badminton players, and all of the participants were voluntarily recruited. Other inclusion criteria were a history of regular participation in international and national competitions and training experience ranging from 7 to 15 years, and no personal history of smoking, cardiovascular disease, diabetes mellitus, and pulmonary disease. The research design and methods were approved by the Institutional Review Board of the University of Taipei, Taiwan (IRB-2019-060, 12 December 2019). Written informed consent was obtained from all participants before the experimental procedures. The data of participants were collected during their off time from training. Figure 1 illustrates the order of the measurements in the present study. Basic anthropometric parameters, including body weight, height, and body mass index (BMI), were recorded. Body weight was measured by physician digital scale (Tanita Corp., Tokyo, Japan), and height was measured using a portable stadiometer (Seca scales, Hamburg, Germany). BMI (kg/m^2^) was calculated by dividing the weight in kilograms by the height in meters squared. Additionally, body composition was determined using dual-energy X-ray absorptiometry (DXA, GE Healthcare Inc., Madison, WI, USA). Bone mineral density (BMD), muscle mass, and fat mass of the total body were obtained from the analyzing software provided by DXA. Moreover, the Z-score represents the bone density, compared with the average bone density of the age-matched adults. It is commonly used for young adults and is helpful in diagnosing osteoporosis.

### 2.2. Measurements of HRV and Arterial Stiffness

Short-term HRV data were recorded for 5 min at rest by using the Polar heart rate monitor (POLAR, RS800CX, Kempele, Finland) and further analyzed using an HRV analysis software program (Nevrokard, Izola, Slovenia). The time- and frequency-domain measures were analyzed by the HRV analysis software [23]. For the time-domain measures, the mean heart rate (HR, ms), mean normal-to-normal (NN) intervals (ms), and standard deviation of the NN intervals (SDNN, ms) was analyzed. For the frequency-domain measures, low-frequency (LF) power (0.04–0.15 Hz), high-frequency (HF) power (0.15–0.4 Hz), and the ratio of LF-to-HF power (LF/HF) were evaluated. During data collection, the participants were asked to lie in the supine position in a quiet, private, semi-darkened, and air-conditioned room (22–24 °C). They were instructed to avoid caffeine and alcohol for 24 h before and not undergo exercise training and intense activities for 48 h before HRV recording. Additionally, they consumed a light meal approximately 2 h before the assessment of HRV. The assessment was conducted for all participants before noon.

After HRV recording, data regarding arterial stiffness were collected using a CardioVision device (MS-2000, Osachi, Nagano, Japan) under the same condition [24]. Systolic blood pressure (SBP), diastolic blood pressure (DBP), pulse pressure (PP), and the arterial stiffness index (ASI) were obtained from the right brachial artery in the resting condition. The ASI was automatically calculated by measuring the blood pressure in the extremities with an oscillometric method. The average of two readings measured separately for 3 min was calculated for each participant.

### 2.3. Measurement of Aerobic Capacity

Aerobic capacity was evaluated using the Bruce incremental load protocol. An incremental exercise test was performed with the treadmill running. The testing system (VIASYS Vmax Series, SensorMedics Corporation, Yorba Linda, CA, USA) comprised a treadmill, a gas analyzer, and an electrocardiographic monitor. The intensity level from 1 to 7 (speed and inclination) was gradually increased every 3 min as follows until the participants were exhausted or fatigued volitionally [25]:

Level 1. Speed: 2.7 km/h, inclination: 10%, lasting 3 min;

Level 2. Speed: 4.0 km/h, inclination: 12%, lasting 3 min;

Level 3. Speed: 5.5 km/h, inclination: 14%, lasting 3 min;

Level 4. Speed: 6.8 km/h, inclination: 16%, lasting 3 min;

Level 5. Speed: 8.0 km/h, inclination: 18%, lasting 3 min;

Level 6. Speed: 8.9 km/h, inclination: 20%, lasting 3 min;

Level 7. Speed: 9.7 km/h, inclination: 22%, lasting 3 min.

During the exercise test, oxygen consumption ($\dot{V}$O_2_), carbon dioxide production, minute ventilation, and the respiratory exchange ratio (RER) were measured breath-by-breath using the testing system. The time to exhaustion was also recorded for each participant. The decisive criterion for assessing $\dot{V}$O_2_max was made when one of the following conditions was achieved: a plateau (<150 mL/min increase) in $\dot{V}$O_2_, RER over 1.10, or heart rate over 90% of the age-predicted heart rate maximum (220-age) [26]. The $\dot{V}$O_2_ at the anaerobic threshold (AT) was determined by the V-slope method during the exercise test. Furthermore, resting blood pressure and blood pressure during the exercise test were continuously recorded using the automated blood pressure monitor (Tango^+^, SunTech Medical Inc., Morrisville, NC, USA).

### 2.4. Statistical Analysis

Data are presented as the mean ± standard deviation. The normality of our data was assessed by the Shapiro–Wilk Test. Accordingly, general characteristics, aerobic capacity, HRV, and arterial stiffness parameters were normally distributed; therefore, the independent *t*-test was used to determine significant differences between male and female participants. Age, muscle mass, training experience, TTE, SDNN, LF/HF, and ASI were not normally distributed, in which case the Mann–Whitney U test was used to compare differences between groups. The effect size by using Hedge’s g between groups was calculated, with small, medium, and large effects being defined as 0.2, 0.5, and 0.8, respectively [27]. Pearson’s correlation analyses were performed to examine the correlation between variables of interest. Statistical significance was set at *p* < 0.05, and the statistical analyses were conducted using the SPSS software (Version 25.0, IBM Corporation, Armonk, NY, USA).

## 3. Results

### 3.1. General Characteristics

A total of 36 elite badminton athletes were included in this study. Height and weight significantly differed between the male and female participants. However, no significant differences in age, BMI, years of training, bone mineral density, and Z-score were noted between the two groups. Furthermore, the female participants exhibited significantly higher fat mass and fat percentage than did the male participants. By contrast, the male participants had significantly higher muscle mass than did the female participants (Table 1). The effect size is indicated in Table 1, according to which large effect sizes for height, weight, fat mass, fat percentage, and muscle mass were observed between sexes.

### 3.2. Aerobic Capacity

Table 2 lists the aerobic capacity obtained from the incremental exercise test with the treadmill running. The male participants exhibited significantly higher $\dot{V}$O_2_max, anaerobic threshold (AT), time to exhaustion (TTE), and maximal SBP (SBPmax) than did the female participants. However, no significant differences in the maximal heart rate (HRmax), maximal DBP (DBPmax), and RER were noted between the groups. The effect size is indicated in Table 2, according to which large effect sizes for $\dot{V}$O_2_max, AT, TTE, and SBPmax were observed between sexes.

### 3.3. HRV and Arterial Stiffness

Table 3 lists the values of HRV and arterial stiffness at rest. No significant differences in HRV measures—namely, the mean HR, mean NN, SDNN, low-frequency power in normalized units (LFnu), high-frequency power in normalized units (HFnu), and the ratio of LF-to-HF power (LF/HF) were observed between the male and female participants. However, the male participants exhibited significantly higher SBP, PP, and ASI under the resting condition than did the female participants. No significant difference in DBP was observed between the groups. The effect size is demonstrated in Table 3. We found small effect sizes for HRV measures and large effect sizes for SBP, PP, and ASI between sexes.

### 3.4. Correlation Analysis

Table 4 represents the correlation analysis of SDNN, LF/HF, and ASI with $\dot{V}$O_2_max in male and female participants. A significant negative correlation between $\dot{V}$O_2_max and SDNN was found in female participants but not in male participants. In addition, no significant correlation between LF/HF and ASI with $\dot{V}$O_2_max existed in both groups.

## 4. Discussion

This study examined the differences between male and female badminton athletes regarding aerobic capacity, HRV, and arterial stiffness. Our findings revealed that the male badminton athletes exhibited a significantly higher $\dot{V}$O_2_max, anaerobic threshold, and time to exhaustion than their female counterparts. By contrast, female badminton athletes showed superior arterial function, compared with male badminton athletes. However, no significant differences between sexes in terms of HRV measures were noted. In addition, the correlation analysis showed that a significant correlation between $\dot{V}$O_2_max and SDNN existed in female badminton athletes, but no other significant correlations were found in both groups.

In agreement with the findings of previous studies, our data showed large significant differences between male and female badminton athletes in relation to $\dot{V}$O_2_max [3,28]. $\dot{V}$O_2_max, which is considered a direct marker of aerobic capacity, tended to be higher in the male participants than in the female participants. Furthermore, in previous Czech [3] and Indian [28] studies of national athletes in badminton and hockey, respectively, $\dot{V}$O_2_max values were higher among men (63.2 ± 3.7 and 55.85 ± 3.94 mL/kg/min, respectively) than women (55.2 ± 2.6 and 43.92 ± 2.76 mL/kg/min, respectively). This sex difference could be attributed to lower body fat percentages and higher muscle mass and hemoglobin levels in men [29,30]. A previous study reported that skeletal muscle mass was positively correlated with $\dot{V}$O_2_max [31]. These findings imply that the amount of muscle mass might be responsible for sex differences in $\dot{V}$O_2_max. Further studies are needed to examine the role of muscle mass contributing to the sex difference in $\dot{V}$O_2_max among elite athletes. Generally, the hemoglobin level of men is approximately 10% higher than that in women [32]. However, whether the hemoglobin level affects the sex difference in $\dot{V}$O_2_max in badminton athletes should be further studied. Furthermore, the results of our study demonstrated large significant differences between male and female badminton athletes with regard to the anaerobic threshold. In agreement with our results, a significantly higher anaerobic threshold in male adolescent cross-country runners than in their female counterparts was observed by Cunningham [33]. Moreover, Yasuda et al. [34] reported a higher anaerobic threshold in recreationally active male participants than in their female counterparts during a leg cycling test.

In the present study, the maximal blood pressure was also determined during the incremental exercise test. We observed a significantly higher maximal SBP in the male participants than in the female participants. In agreement with our study, Caselli et al. noted a significantly higher maximal SBP in men than in women, through an examination of SBP in response to maximal exercise among a cohort of 1876 young normotensive elite athletes [35]. Wheatley et al. [36] observed lower SBP in response to maximal and submaximal exercise in women than in men. These findings could be attributed to the inability to elevate stroke volume through an increase in the heart rate, the attenuated sympathetic response, and a higher basal vasodilatory state in females.

In this study, we noted large significant differences between male and female badminton athletes regarding arterial stiffness; this finding was in accordance with those reported by Doonan et al. [37] and Perdomo et al. [38]. Those two studies have observed significantly higher levels of arterial stiffness at rest, indicated by pulse wave velocity, in healthy young men than in their female counterparts. In addition, Nieman et al. noted differences in arterial stiffness following 2 h of running between male and female trained runners [39]. These results might be attributed to sex hormones and differences in endothelin-1 production between sexes [40,41]. Estrogen exerts cardioprotective effects, and sex hormones and their receptors might partially regulate sex differences in cardiovascular outcomes [42]. Some studies have speculated that arterial stiffness was decreased in women and increased in men after sexual maturity [42,43,44]. This is consistent with the age (20 to 28 years old) of our recruited badminton athletes. In addition, strength training for increasing muscle mass has been associated with lower arterial compliance, which refers to higher arterial stiffness [45]. Previous studies demonstrated that young adults participating in strength-based sports, which increased muscle mass, would exhibit higher arterial stiffness, compared with sedentary individuals [46,47]. However, whether higher muscle mass affects the sex difference in terms of arterial stiffness in badminton athletes should be further confirmed in future studies.

The results of our study revealed significantly higher resting SBP in the male participants than in the female counterparts. Similarly, Žemva et al. noted significantly higher values of SBP in male dancers than in female dancers [48]. In addition, Caselli et al. examined the resting SBP and DBP in Olympic athletes and found significantly higher values of SBP and DBP in men than in women [35]. Although we observed large significant differences in SBP between male and female participants, no significant differences in DBP were observed between the groups in the present study. The major determinants underlying these differences in blood pressure between male and female participants include the angiotensin system, the sympathetic nervous activity, sex hormones, ET-1, and the immune system [49]. Furthermore, a study examined PP in young healthy Swedish adults and found significantly higher values in men than in women, which is in agreement with the results from our study [50]. The major factors responsible for differences in PP are arterial stiffness and cardiac output [51].

In the present study, HRV parameters did not significantly differ between male and female badminton athletes. By contrast, Hedelin et al. [52] observed a higher level of parasympathetic activity, indicated by higher HF and total power, in female adolescent cross-country skiers than in their male counterparts. Berkoff et al. [53] found significant differences in the LFnu, the LF-to-HF ratio, and the percentage of successive NN intervals that differ by more than 50 ms (pNN50) between elite male and female track-and-field athletes. In their study, LF power was significantly lower in women and both the LF-to-HF ratio and pNN50 were significantly higher in women than in men. Previous studies [54,55] have reported that men exhibited a more blunted parasympathetic modulation of cardiovascular activity, compared with women. Koenig and Thayer [55] stated that these variations in autonomic modulation might be attributed to differences in the levels of estrogen, oxytocin, and neural control between men and women. By contrast, Schäfer et al. [56] found no sex differences in HRV at rest in the supine position in elite cross-country skiers. The authors speculated that these findings could be explained by a very high level of training experience of participants and might be due to the inability to further increase HRV. Similarly, the participants examined in our study were elite athletes, which could have contributed to the absence of sex differences in HRV measures.

Sex differences also existed in our correlation analysis. We found a significant negative correlation between $\dot{V}$O_2_max and SDNN in female participants but not in male participants. Additionally, no other relationships existed in both groups. Previous studies indicated that some HRV indicators, such as higher SDNN and pNN50, were significantly correlated with greater $\dot{V}$O_2_max [57,58]. Some researchers suggested that correlations between HRV indicators and $\dot{V}$O_2_max existed mainly due to the relationship between heart rate and $\dot{V}$O_2_max. Possible reasons explaining our contrasting results might be the training status of our participants (i.e., elite badminton players) and smaller sample size. Further research should be conducted to verify the relationship between HRV indicators and $\dot{V}$O_2_max in badminton athletes, and the underlying mechanisms need more exploration in future studies.

There were some limitations in our study. First, this study had a relatively small number of participants, which would limit the generalization of our results. Therefore, the recruitment of more participants would be preferable in future research. Second, sex differences in aerobic capacity and arterial stiffness in elite badminton athletes were observed in this study. However, the physiological mechanisms responsible for these sex differences remain unidentified. Future research should be conducted to identify the potential factors affecting these sex differences in cardiovascular modulation. Lastly, the short-term recordings were used to evaluate the HRV measures, and no sex differences in HRV were found in elite badminton athletes. The 24 h long-term recordings (e.g., electrocardiography) for the HRV measures, which provide other parameters (e.g., SDNN index and pNN50), are recommended to further confirm and examine these results in the athletes.

## 5. Conclusions

Our study revealed sex differences in the performance of elite badminton athletes with respect to aerobic capacity and arterial stiffness. However, no significant differences between sexes on HRV measures were observed. In addition, the correlation analysis showed that a significant correlation between $\dot{V}$O_2_max and SDNN existed in female badminton athletes, but no other significant correlations were found in both groups. These findings in terms of sex differences could provide more information in either health promotion or sports performance when designing appropriate intervention programs for improving cardiovascular function. Furthermore, this study may contribute to further understanding of the influences of sex differences on cardiovascular modulation.

## Figures and Tables

**Table 1:** General characteristics.

Variables	Male (*n* = 20)	Female (*n* = 16)	ES	*p* Value
Age (years)	21.0 ± 1.8	21.2 ± 2.3	0.10	0.809
Height (cm)	174.25 ± 6.24	165.21 ± 3.95 *	1.69	<0.001
Weight (kg)	69.75 ± 7.44	62.56 ± 6.71 *	1.01	0.005
BMI (kg/m^2^)	22.92 ± 1.77	22.91 ± 2.17	0.01	0.982
Fat mass (kg)	10.75 ± 3.59	15.62 ± 4.20 *	1.26	0.001
Fat (%)	15.14 ± 4.14	24.59 ± 4.30 *	2.24	<0.001
Muscle mass (kg)	56.06 ± 5.74	44.29 ± 3.47 *	2.42	<0.001
BMD (g/cm^2^)	1.34 ± 0.09	1.29 ± 0.11	0.50	0.161
Z-score	2.08 ± 0.82	2.29 ± 0.99	0.23	0.484
Training experience (years)	10.85 ± 2.08	12.38 ± 2.78	0.63	0.073

Values are presented as the mean ± standard deviation. Abbreviations: BMI, body mass index; BMD, bone mineral density; ES, effect size. * *p* < 0.05, significant differences between male and female participants.

**Table 2:** Sex differences in aerobic capacity.

Variables	Male (*n* = 20)	Female (*n* = 16)	ES	*p* Value
HRmax (bpm)	187.35 ± 11.38	181.25 ± 13.85	0.49	0.156
SBPmax (mmHg)	210.55 ± 24.85	173.06 ± 17.88 *	1.70	<0.001
DBPmax (mmHg)	66.20 ± 16.10	63.25 ± 10.54	0.21	0.532
VO_2_max (ml/kg/min)	60.38 ± 8.98	48.13 ± 7.72 *	1.45	<0.001
AT (ml/kg/min)	41.50 ± 7.26	32.51 ± 6.19 *	1.32	<0.001
RER	1.13 ± 0.07	1.14 ± 0.11	0.11	0.891
TTE (sec)	902.15 ± 120.15	780.56 ± 67.63 *	1.21	<0.001

Values are presented as the mean ± standard deviation. Abbreviations: HRmax, maximal heart rate; SBPmax, maximal systolic blood pressure; DBPmax, maximal diastolic blood pressure; $\dot{V}$O_2_max, maximal oxygen consumption; AT, oxygen consumption at the anaerobic threshold; RER, respiratory exchange ratio; TTE, time to exhaustion; ES, effect size. * *p* < 0.05, significant differences between male and female participants.

**Table 3:** Sex differences in HRV and arterial stiffness at rest.

Variables	Male (*n* = 20)	Female (*n* = 16)	ES	*p* Value
HRV				
Mean HR (bpm)	58.62 ± 10.49	56.04 ± 7.85	0.27	0.419
Mean NN (ms)	1063.95 ± 207.50	1097.06 ± 154.33	0.18	0.599
SDNN (ms)	65.37 ± 40.82	62.38 ± 38.01	0.08	0.726
LFnu	16.92 ± 3.77	15.82 ± 4.21	0.28	0.413
HFnu	42.11 ± 6.27	42.99 ± 8.21	0.12	0.717
LF/HF	0.45 ± 0.16	0.42 ± 0.18	0.18	0.524
Arterial stiffness				
SBP (mmHg)	125.27 ± 7.76	107.16 ± 11.09 *	1.93	<0.001
DBP (mmHg)	61.58 ± 5.84	57.74 ± 7.98	0.56	0.105
PP (mmHg)	63.37 ± 8.04	49.54 ± 7.22 *	1.80	<0.001
ASI	63.56 ± 12.55	53.83 ± 8.03 *	0.90	0.016

Values are presented as the mean ± standard deviation. Abbreviations: HR, heart rate; NN, normal-to-normal interval; SDNN, standard deviation of the NN intervals; LFnu, low-frequency power in normalized units; HFnu, high-frequency power in normalized units; LF/HF, the ratio of LF-to-HF power; SBP, systolic blood pressure; DBP, diastolic blood pressure; PP, pulse pressure; ASI, arterial stiffness index; ES, effect size. * *p* < 0.05, significant differences between male and female participants.

**Table 4:** Correlation analysis of SDNN, LF/HF, and ASI with $\dot{V}$O_2_max.

Groups	$\dot{V}$O_2_max-SDNN	$\dot{V}$O_2_max-LF/HF	$\dot{V}$O_2_max-ASI
Male (*n* = 20)	0.388 (*p* = 0.091)	−0.130 (*p* = 0.583)	0.282 (*p* = 0.228)
Female (*n* = 16)	−0.532 * (*p* = 0.034)	0.104 (*p* = 0.702)	−0.169 (*p* = 0.532)

Values are presented as coefficient of correlation r and level of statistical significance. (* *p* < 0.05). Abbreviations: $\dot{V}$O_2_max, maximal oxygen consumption; SDNN, standard deviation of the NN intervals; LF/HF, the ratio of LF-to-HF power; ASI, arterial stiffness index.

## Data Availability

The data presented in this study are available from the corresponding author on reasonable request.
